# Nonlinear stability analysis of whirl flutter in a rotor-nacelle system

**DOI:** 10.1007/s11071-018-4472-y

**Published:** 2018-08-02

**Authors:** Christopher Mair, Djamel Rezgui, Branislav Titurus

**Affiliations:** Department of Aerospace Engineering, University of Bristol, Queens Building, Bristol, UK

**Keywords:** Bifurcations, Continuation, Stability boundary, Nonlinear stiffness, Whirl flutter

## Abstract

Whirl flutter is an aeroelastic instability that affects propellers/rotors and the surrounding airframe structure on which they are mounted. Whirl flutter analysis gets progressively more complicated with the addition of nonlinear effects. This paper investigates the impact of nonlinear pylon stiffness on the whirl flutter stability of a basic rotor-nacelle model, compared to a baseline linear stiffness version. The use of suitable nonlinear analysis techniques to address such a nonlinear model is also demonstrated. Three types of nonlinearity were investigated in this paper: cubic softening, cubic hardening and a combined cubic softening—quintic hardening case. The investigation was conducted through a combination of eigenvalue and bifurcation analyses, supplemented by time simulations, in order to fully capture the effects of nonlinear stiffness on the dynamic behaviour of the rotor-nacelle system. The results illustrate the coexistence of stable and unstable limit cycles and equilibria for a range of parameter values in the nonlinear cases, which are not found in the linear baseline model. These branches are connected by a number of different bifurcation types: fold, pitchfork, Hopf, homoclinic and heteroclinic. The results also demonstrate the importance of nonlinear whirl flutter models and analysis methods. Of particular interest are cases where the dynamics of the nacelle are unstable despite linear analysis predicting stable behaviour. A more complete stability envelope for the combined model was generated to take account of this phenomenon.

## Introduction

The aeroelastic instability known as whirl flutter is an important consideration in aircraft design. A propeller or rotor mounted in a wing nacelle may be susceptible to whirl flutter. Typically associated with tiltrotors and some fixed wing aircraft, the phenomenon is manifested in the hub whirling around its original position. Aerodynamic forces acting on the blades and gyroscopic effects acting on the rotor as a whole couple with wing structural modes to produce this unstable motion, which can damage or even destroy the aircraft structure [1]. Two whirl flutter modes exist—forward whirl (FW) and backward whirl (BW)—identified by the sense of the whirl relative to the rotor’s rotation; forward denotes that the whirl and the rotor are spinning in the same direction. With their large and flexible blades, tiltrotors are particularly susceptible to whirl flutter. For given cruise speed requirements, whirl flutter stability considerations impact the design of their wings, pylons and rotors [2]. Designing to prevent whirl flutter becomes more complicated in the presence of nonlinearity. Nonlinearities are often neglected for convenience in modelling, contingent on the applicability of some assumption(s) given the scope of a particular investigation, though aerospace structures regularly exhibit nonlinear behaviours [3]. Furthermore, there is little mention in the existing literature of nonlinear whirl flutter studies being conducted.

The current literature has investigated methods of improving stability margins by alterations to existing rotor designs [4] and studied the impact of effects such as control system stiffness [5]. However, in almost all cases, these studies restricted the structural modelling to linear approximations, which is contingent on the assumption of small deformations. Various kinds of nonlinearity have been shown to have a non-negligible effect on system behaviour. A review of the impact of various types of structural nonlinearity on system dynamics was provided by Breitbach [6], with further specific investigations conducted by Dowell and Ilgamov [7]. In both cases, analytical frameworks and the effects of each nonlinearity on flutter predictions are suggested. Masarati et al. [8] showed that nonlinear effects at the blade level can ultimately affect overall stability in a tiltrotor system. Moreover, Krueger [3] showed that nonlinearities introduced by the influence of the drivetrain, free-play and backlash can create a behavioural discrepancy between rotors in windmill and thrust mode. While the main focus of Krueger’s paper is to present a multibody modelling approach of a previous wind tunnel test performed as part of the European ADYN project, the effects of nonlinearity were investigated through the introduction of nonlinear springs in the computational model. Spring stops were also added to provide hard limits on model deflection, and a good agreement with the wind tunnel test data was shown. Considering the repeated demonstration that nonlinear effects have an impact on a system’s behaviour, they are therefore an important modelling consideration.

Park et al. [9] investigated whirl flutter with a nonlinear structural model, though the focus of the paper was an overall design optimisation framework as opposed to any impacts on the whirl flutter predictions made by using nonlinear elements in the model. Additionally, the stability analysis was conducted through time simulations alone rather than any dedicated direct method such as bifurcation analysis. That is, the whirl flutter onset speed was determined through iterative time simulations to find the maximum airspeed that did not result in flutter. Furthermore, investigations by Janetzke et al. [10] used nonlinear aerodynamic models adapted from aerofoil data, though the structural aspects of the model did not appear to have benefitted from the same approach.

Moreover, Lee and Tron [11] showed that the inclusion in a model of certain known nonlinear effects in control surfaces can lead to the early onset of flutter behaviours. A linear model incorrectly predicts a much higher onset speed.

Nonlinearity between load and displacement in a structure’s stiffness may be caused by non-uniformity in either geometry or material properties. Both sources of non-uniformity are likely to be present in any aerospace structure, meaning that linear approximations of stiffness are only acceptable when deflections are very small. Realistic spring characteristics may include both softening and hardening phenomena at different points in the stiffness profile, visible as decreases and increases, respectively, in the gradient of the stress–strain curve for a given spring structure [12]. Use of cubic terms for more representative stiffness modelling at large deflections can be found in Thompson [13].

The previous studies either stopped short of a fully nonlinear analysis or avoided using nonlinear models altogether. In order to understand the effect of nonlinear model aspects on a system’s behaviour, suitable analysis methods must be used, namely continuation and bifurcation methods. Such methods have so far been applied in only a small number of rotorcraft dynamical problems, specifically flight mechanics [14], ground resonance [15] and rotor vortex ring state [16], though their inclusion in rotary-wing studies is steadily becoming more prevalent as they are powerful in solving problems such as the identification of instability scenarios of rotor blades [17]. Continuation methods were also used in the AW159/Wildcat Release to Service document [18, 19], to investigate free-play effects on the behaviour of the tail rotor. Salles et al. [20] used continuation and bifurcation methods to investigate bifurcations in the behaviour of whole engine rotordynamic models, due to the presence of nonlinearities, although their analysis was conducted in the frequency domain.

In this paper, a basic whirl flutter system is presented in Sect. 2. This model included linear and subsequently nonlinear expressions for yaw stiffness, specifically polynomial terms proportional to the cube and fifth power of displacement. Section 3 describes the stability analysis methods used, and these are applied to the linear and nonlinear models as appropriate. The analysis was carried out for a number of cases to study the effects of nonlinearity for a set of selected parameters. The stability results and bifurcation diagrams generated are discussed in Sect. 4, along with revised stability boundaries that take account of the additional effects from the nonlinearities introduced.

## Whirl flutter model

**Fig. 1:**
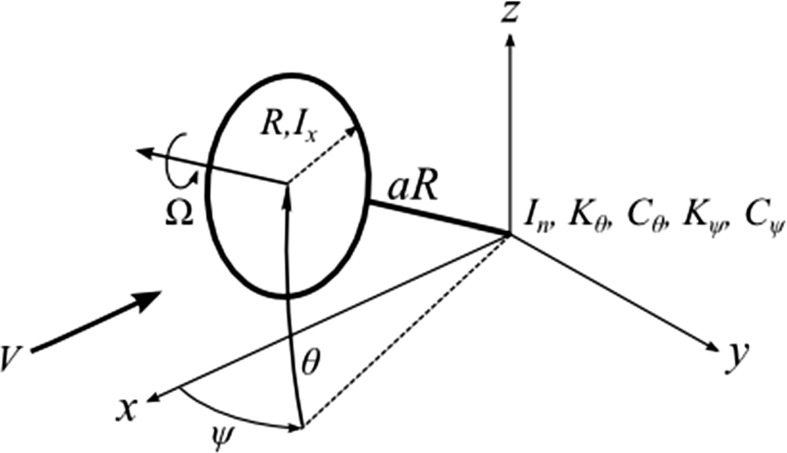
Whirl flutter model schematic diagram

A basic model given by Bielawa [21] and originally formulated by Reed [22] was used. In this model, a rotor of radius *R*, spinning at angular speed $\Omega$ with moment of inertia about its rotational axis $I_{x}$, is able to oscillate in pitch $\theta$ and yaw $\psi$ about an effective pivot point with moment of inertia $I_{n}$. The dynamical contributions of the wing structure are modelled with equivalent stiffness *K* and damping *C* properties in the pitching and yawing directions at the effective pivot point, to which the rotor is connected at a distance of *a* multiples of its radius. The rotor is subjected to the axial flow condition: a freestream velocity *V* is incident on the rotor system along the *x* axis. The system schematic is shown in Fig. 1.

The original model in Reed [22] features linear structural stiffness properties and was used as a baseline for comparison with the nonlinear stiffness versions. The equations of motion governing the system, as given by Bielawa [21], are stated in Eq. (1).

1 $$\begin{aligned}&\left[ {{\begin{array}{cc} {I_n }&{} 0 \\ 0&{} {I_n } \\ \end{array} }} \right] \left[ {{\begin{array}{c} {\ddot{\theta }} \\ {\ddot{\psi }} \\ \end{array} }} \right] +\left[ {{\begin{array}{cc} {C_\theta }&{} {-I_x \Omega } \\ {I_x \Omega }&{} {C_\psi } \\ \end{array} }} \right] \left[ {{\begin{array}{c} {\dot{\theta }} \\ {\dot{\psi }} \\ \end{array} }} \right] \nonumber \\&\quad +\left[ {{\begin{array}{cc} {K_\theta }&{} 0 \\ 0&{} {K_\psi } \\ \end{array} }} \right] \left[ {{\begin{array}{c} \theta \\ \psi \\ \end{array} }} \right] =\left[ {{\begin{array}{c} {M_\theta } \\ {M_\psi } \\ \end{array} }} \right] \end{aligned}$$

where $M_{\theta }$ and $M_{\psi }$ are aerodynamic moments in pitch and yaw, respectively, and are defined in Eqs. (2) and (3). They were derived in the manner employed in Ribner’s work [23] on forces and moments generated by propellers experiencing yaw and yawing rates at their hub. Ribner’s derivation is founded upon blade element theory and assumes quasi-steady aerodynamics, an aspect that some investigations, such as that by Kim et al. [24], have built upon. A key aspect of Ribner’s work that separated it from existing theory at the time was the inclusion of induction/inflow effects, “analogous to the downwash associated with a finite wing”. It can be seen from the equations that there is coupling only at the angular displacement level.

2 $$\begin{aligned} M_\theta= & {} \frac{N_B }{2}K_a R\left[ -( {A_3 +a^{2}A_1 } )\frac{\dot{\theta }}{\Omega } \right. \nonumber \\&\left. -{A}'_2 \psi +a{A}'_1 \theta \right] \end{aligned}$$

3 $$\begin{aligned} M_\psi= & {} \frac{N_B }{2}K_a R\left[ -( {A_3 +a^{2}A_1 } )\frac{\dot{\psi }}{\Omega } \right. \nonumber \\&\left. +{A}'_2 \theta +a{A}'_1 \psi \right] \nonumber \\ K_a= & {} \frac{1}{2}\rho c_{l,\alpha } R^{4}\Omega ^{2} \end{aligned}$$

**Fig. 2:**
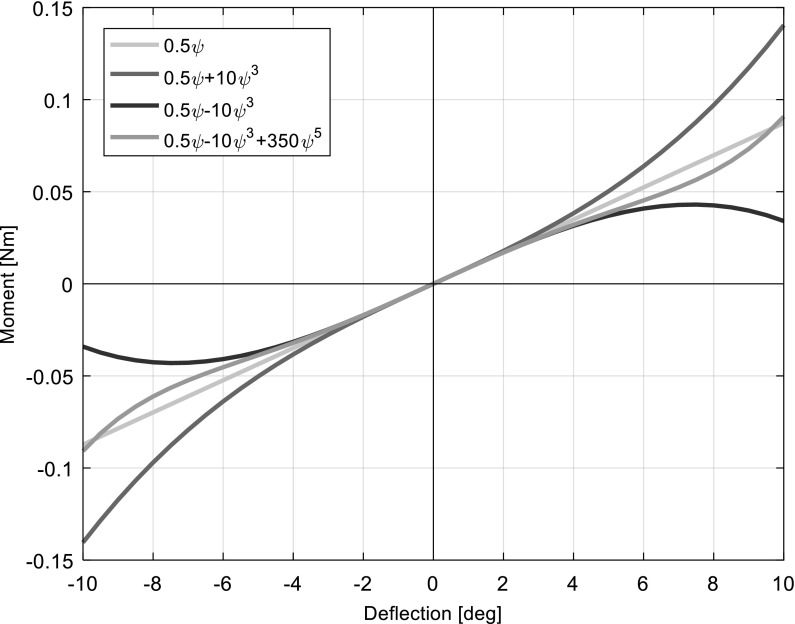
Nonlinear stiffness profiles used

where $K_{a}$ is a consolidation of terms for more concise presentation; $\rho$ denotes air density and $c_{l,\alpha }$ denotes the blade section lift slope. The $A_{i}$ terms are aerodynamic integrals that arise from integrating lift along each blade and summing the contributions from each, and are defined as:

4 $$\begin{aligned}&A_1 =\frac{c}{R}\int _0^1 {\frac{\mu ^{2}}{\sqrt{\mu ^{2}+\eta ^{2}}}d\eta } \end{aligned}$$

5 $$\begin{aligned}&{A}'_1 =\mu A_1 \end{aligned}$$

6 $$\begin{aligned}&{A}'_2 =\frac{c}{R}\int _0^1 {\frac{\mu ^{2}\eta ^{2}}{\sqrt{\mu ^{2}+\eta ^{2}}}d\eta } \end{aligned}$$

7 $$\begin{aligned}&A_3 =\frac{c}{R}\int _0^1 {\frac{\eta ^{4}}{\sqrt{\mu ^{2}+\eta ^{2}}}d\eta } \end{aligned}$$

8 $$\begin{aligned}&\mu =\frac{V}{\Omega R} \end{aligned}$$

The $A_{2}$ (without a hyphen) integral features only in the derivation of these expressions [21]; however, the original nomenclature has been retained here. For the nonlinear cases, the original linear expression for the structural yaw stiffness (i.e. $K_{\psi }\psi$) was replaced with a polynomial of the form given in Eq. (9), where “nl” denotes “nonlinear”. Here, the stiffness is a function of angular deflection. The influence of each term is controlled via dedicated coefficients $K_{i}$. As the pitch and yaw degrees of freedom in the original formulation were modelled in exactly the same way, either could have been selected for nonlinear adaption without any qualitative impact on the results.

9 $$\begin{aligned} K_{\psi ,nl} \left( \psi \right) \psi= & {} K_1 \psi +K_2 \psi ^{3}+K_3 \psi ^{5} \nonumber \\= & {} ( {K_1 +K_2 \psi ^{2}+K_3 \psi ^{4}} )\psi \end{aligned}$$

The nonlinear stiffness expression can provide softening behaviours by using negative values of $K_{2}$ and/or $K_{3}$, and hardening behaviours by using positive values. The cubic term is dominant at smaller deflections, while the quintic term is dominant at larger deflections, allowing both softening and hardening behaviours to be observed in the same stiffness profile if $K_{2}$ and $K_{3}$ have opposite signs. In order to reflect the most prevalent types of nonlinear spring stiffness, this research selected the following three stiffness profiles for investigation: cubic hardening ($K_{2}=10~\hbox {Nm rad}^{-3}$, $K_{3}=0~\hbox {Nm rad}^{-5})$, cubic softening ($K_{2}=-10~\hbox {Nm rad}^{-3}$, $K_{3}=0~\hbox {Nm rad}^{-5}$) and combined cubic softening—quintic hardening ($K_{2}= -10~\hbox {Nm rad}^{-3}$, $K_{3}=350~\hbox {Nm rad}^{-5}$). The linear coefficient $K_{1}$ was varied between $-\,0.3$ and $0.5~\hbox {Nm rad}^{-1}$ as the independent variable in each case. The overall shape of these profiles compared to the original linear model is shown in Fig. 2. Hereafter, the model employing the original linear yaw stiffness expression is referred to as the “linear model”, and the models employing the nonlinear stiffness expressions as the “hardening model”, “softening model” or “combined model” as appropriate.

The model equations were written in state-space form, as shown in (10) and (11):

10 $$\begin{aligned} \dot{\mathbf{Y}}=f\left( {\mathbf{Y},\mathbf{p}} \right) ,\quad \mathbf{Y}\in \mathfrak {R}^{4},\quad \mathbf{p}\in \mathfrak {R}^{n} \end{aligned}$$

and

11 $$\begin{aligned} \mathbf{Y}=\left[ {{\begin{array}{c} \theta \\ \psi \\ {\dot{\theta }} \\ {\dot{\psi }} \\ \end{array} }} \right] \end{aligned}$$

where **Y** is the state vector and **p** is a vector of *n* parameters. The model was implemented in MATLAB 2016a [25], and time simulations were generated using the ode45 solver, which implements an explicit Runge–Kutte (4, 5) formula, the Dormand–Prince pair [26]. The parameter values used throughout the investigation (Table 1) were retained, where possible, from Reed. Where ranges of parameters were used in Reed [22], the midpoint value was taken for this parameter set. These values represent a scaled wind tunnel rotor-nacelle system; however, the qualitative results achieved from the following analyses are applicable to full size aircraft.

**Table 1:** Datum parameter values

Description	Symbol	Value
Rotor radius	*R*	0.152 m
Rotor angular velocity	$\Omega$	$40~\hbox {rad s}^{-1}$
Freestream velocity	*V*	$6.7~\hbox {ms}^{-1}$
Pivot length to rotor radius ratio	*a*	0.25
Rotor moment of inertia	$I_{x}$	$0.000103~\hbox {kg m}^{2}$
Nacelle moment of inertia	$I_{n}$	$0.000178~\hbox {kg m}^{2}$
Structural pitch damping	$C_{\theta }$	$0.001~\hbox {Nm s rad}^{-1}$
Structural pitch stiffness	$K_{\theta }$	$0.4~\hbox {Nm rad}^{-1}$
Structural yaw damping	$C_{\psi }$	$0.001~\hbox {Nm s rad}^{-1}$
Structural yaw stiffness	$K_{\psi }$	$0.4~\hbox {Nm rad}^{-1}$
Number of blades	$N_{B}$	4
Blade chord	*c*	0.026 m
Blade lift slope	$c_{l,\alpha }$	$2\pi ~\hbox {rad}^{-1}$

## Stability analysis methods

Initially, eigenvalue analysis was used to assess the stability of the linear system. This standard method places the equations of motion of the system in state-space form in order to obtain the Jacobian matrix ***J*** about an equilibrium point, defined as

12 $$\begin{aligned} \dot{\mathbf{Y}}=\mathbf{JY} \end{aligned}$$

where **Y** is the state vector, which for the whirl flutter model used in this paper is defined in Eq. (11). If the various terms in the aerodynamic moment expressions [Eqs. (2) and (3)] are brought over to the left-hand side of the equation and incorporated into the relevant matrices, the equations of motion assume the form of

13 $$\begin{aligned}&{} \mathbf{M}\ddot{\mathbf{X}}+\mathbf{C}\dot{\mathbf{X}}+\mathbf{KX}=\mathbf{0} \nonumber \\&{} \mathbf{X}=\left[ {{\begin{array}{c} \theta \\ \psi \\ \end{array} }} \right] \end{aligned}$$

and therefore the Jacobian matrix for this system is

14 $$\begin{aligned} \mathbf{J}=\left[ {{\begin{array}{cc} \mathbf{0}&{} \mathbf{I} \\ {-\mathbf{M}^{-\mathbf{1}}{} \mathbf{K}}&{} {-\mathbf{M}^{-\mathbf{1}}{} \mathbf{C}} \\ \end{array} }} \right] \end{aligned}$$

where **0** and **I** are $2\times 2$ zero and identity matrices, respectively. The eigenvalues of the Jacobian matrix contain information about the frequency and damping/decay rate (i.e. stability) of the system’s vibrational modes, and the corresponding right eigenvectors contain the mode shapes. The undamped natural frequency $\omega$ and damping ratio $\zeta$ for a given mode are calculated from the real and imaginary parts of its eigenvalue $\lambda$ using Eqs. (15) and (16). The eigenvalue analysis was also completed in MATLAB.

15 $$\begin{aligned} \omega= & {} \sqrt{\hbox {Re}\left( \lambda \right) ^{2}+\hbox {Im}\left( \lambda \right) ^{2}} \end{aligned}$$

16 $$\begin{aligned} \zeta= & {} \frac{-\hbox {Re}\left( \lambda \right) }{\omega } \end{aligned}$$

For nonlinear systems, numerical continuation and bifurcation theory are used. Given a known solution as a starting point, continuation calculates the steady-state solutions of a dynamical system as one of its parameters, called the continuation parameter, is varied [27]. The computed solutions construct a number of branches that can be either stable or unstable. To determine their stability, either an eigenvalue or Floquet analysis is carried out at each computed solution point, depending on the nature of the solution. For equilibria (also known as fixed points), an eigenvalue analysis can be used—requiring local linearisation in the case of a nonlinear system—whereas periodic solutions (formally limit cycle oscillations, abbreviated to LCO) require Floquet theory to determine their stability [28].

**Fig. 3:**
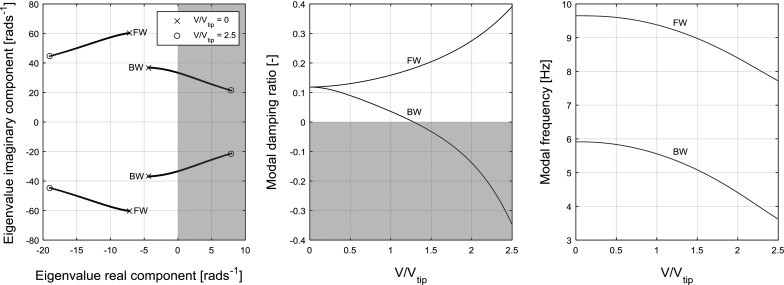
Example frequency (left) and modal damping (right) plots for a sweep in freestream velocity *V* in the linear model. The shaded area is unstable. “FW” denotes the forward whirl mode, while “BW” denotes the backward whirl mode

A bifurcation is a qualitative point change in the system behaviour as a parameter is varied. In other words, when the stability of a system is changed or lost, the system bifurcates. The points at which these stability changes happen are called bifurcation points. Another way to visualise this change is to consider the phase portraits of the system either side of the bifurcation: they are topologically different and therefore one cannot be mapped to the other through a continuous one-to-one transformation [27]. When the system is nonlinear, new solution branches may emerge from the bifurcation points, leading to the presence of multiple solutions for a given set of system parameters. The identification of these different solution branches helps to uncover the global dynamics of the system. Of particular interest are instances where stability is dependent on the magnitude of a perturbation, a hallmark phenomenon of nonlinear systems.

Each type of system (linear, cubic softening etc.) was analysed using the appropriate method. Bifurcation diagrams were produced using the Dynamical Systems Toolbox for MATLAB by Coetzee [29], which uses an implementation of AUTO-07P [30]. Time simulations were also used in both cases to corroborate the predictions of the stability methods.

## Results and discussion

### Linear stability

The eigenvalue analysis described in Sect. 3 allows the stability of a linear system to be quantified in terms of margin, and the cause of any instability encountered to be recognised through the location and movement of the eigenvalues on the complex plane. The eigenvalues, damping ratio and frequency of the linear model’s modes are shown in Fig. 3 as the freestream velocity *V* is swept across a range of values. The two whirl flutter modes described in Sect. 1 were identified through inspection of the eigenvector components. The middle plot shows that the system is predicted to encounter whirl flutter at a $V{/}V_{\mathrm{tip}}$ value of approximately 1.25. The remaining parameter values used are those presented in Table 1.

The concept of a stability boundary diagram between two parameters can be useful for understanding a system’s sensitivity to changes in those parameters, particularly parameters that are readily controllable in the design phase of a practical system, such as an aircraft. Such a diagram can be produced from a grid of the combinations of different values for each parameter. The Jacobian matrix is calculated at each point, and a surface is overlaid where the level is determined by the maximum real component of the Jacobian’s eigenvalues at each point. As the sign of the real component of an eigenvalue determines the stability of the corresponding mode—positive being unstable—and only one unstable eigenvalue is required for overall system instability, a horizontal plane cut of this surface at the level 0 will produce a contour that denotes the boundary between the stable and unstable regions of the parameter grid.

**Fig. 4:**
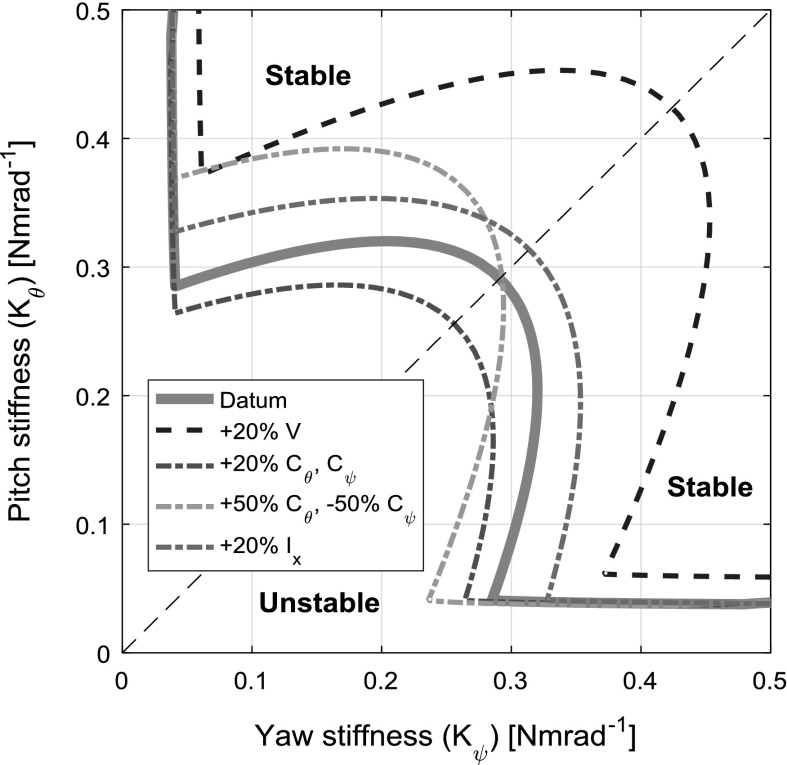
Stability boundary for the linear model in the pitch stiffness and yaw stiffness plane

One such stability boundary that uses parameters that are controllable in the design phase of a rotary-wing aircraft’s rotor system is that between two structural properties: yaw stiffness $K_{\psi }$ and pitch stiffness $K_{\theta }$, shown in Fig. 4. To demonstrate the respective impacts of variations in some of the other physical system parameters, the same stability boundary is plotted for a number of such changes. Increasing the freestream velocity *V* or the rotor’s moment of inertia $I_{x}$ enlarges the unstable region symmetrically, whereas increasing the damping in both degrees of freedom $(C_{\theta },\,C_{\psi })$ diminishes the unstable region symmetrically. Altering the damping parameters asymmetrically enlarges the stable unstable region in the direction of the reduced parameter and reduces it in the direction of the increased parameter. The datum case, using the parameter values given in Table 1, is similar to that achieved by Reed [22].

### Bifurcation analysis

Figure 4 can also be generated by continuation methods, as the system has an equilibrium at $\mathbf{Y}= [0;\,0;\,0;\,0]$ that can be used as a starting solution. Generating the stability boundary this way in fact affords deeper insight than the contour cut method described in Sect. 4.1. Key bifurcation types that are relevant to understanding the behaviour of this rotor-nacelle system, particularly when the nonlinear stiffness profiles are introduced, are Hopf bifurcations, branch points and fold bifurcations. At a Hopf bifurcation, the stability of a fixed point (i.e. an equilibrium) changes, and a periodic solution arises, caused by a pair of complex conjugate eigenvalues crossing the complex plane imaginary axis. At a branch point, the solution changes stability, caused by a single eigenvalue crossing over the complex plane imaginary axis. Because the branch points in this system are of the pitchfork type, two additional equilibrium branches emanate from the bifurcation point. At a fold bifurcation (also known as a limit point), a solution branch changes direction within the solution space and changes stability [27].

Two further bifurcation types that are also observed in the model’s behaviour are the homoclinic bifurcation and the heteroclinic bifurcation. These are global bifurcations that concern the collision of branches. A heteroclinic trajectory is a path in the phase space that connects two equilibria, while a homoclinic trajectory joins a single equilibrium to itself. Heteroclinic and homoclinic bifurcations are points where a limit cycle makes contact with an equilibrium branch at some point along itself, creating a heteroclinic/homoclinic trajectory and annihilating the periodic solutions branch of which it is part. These bifurcations are covered in greater detail in Sect. 4.6. For more information on the subject, the reader is referred to [27, 31].

**Fig. 5:**
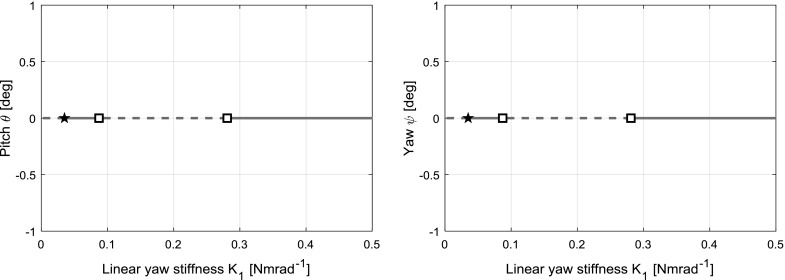
Bifurcation diagram for $K_{\theta }=0.3$, for pitch $\theta$ (left) and yaw $\psi$ (right) with $K_{\psi }$ as the continuation parameter

Choosing $K_{\theta }=0.3$ so that a continuation in $K_{\psi }$ will intersect the regions of interest in the contour-based stability boundary shown in Fig. 4, the bifurcation diagrams shown in Fig. 5 are obtained. In these diagrams, fixed point solutions in pitch $\theta$ and yaw $\psi$ are plotted against the continuation parameter, termed pitch and yaw “projections”, respectively. A key to the symbols and lines used in the bifurcation diagrams shown in this paper is given in Table 2. Particular attention is drawn to periodic solution branches: it is common practice to indicate a branch by the maximum positive extent of the limit cycle at each point.

**Fig. 6:**
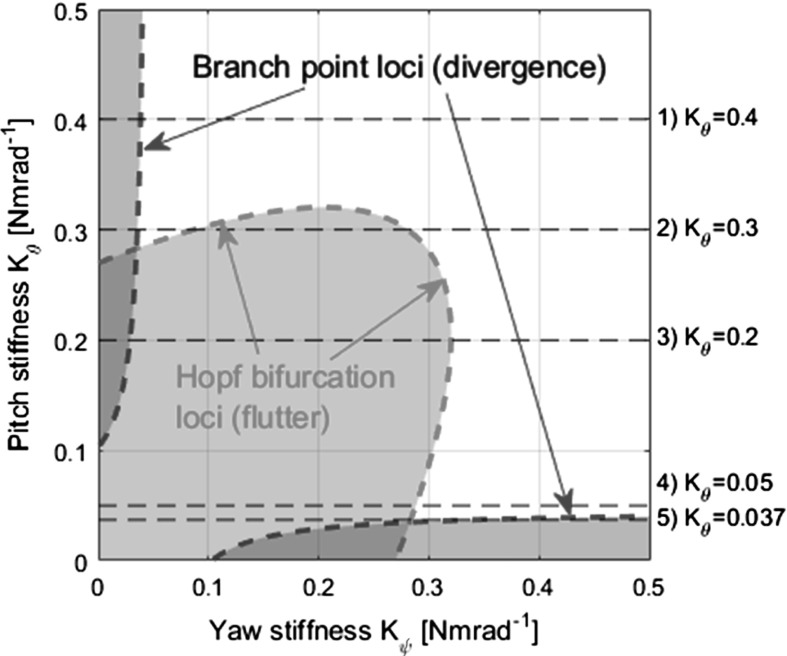
Stability boundary generated by two-parameter continuation. All shaded areas are unstable

Note the Hopf bifurcations (square icon) at $K_{\psi }=0.28$ and $K_{\psi }=0.08$, and the branch point (star icon) at $K_{\psi }=0.03$. The bifurcations are visible at the same points in the projections of $\theta$ and $\psi$. The same bifurcation diagram shown in Fig. 5 can be generated for different values of $K_{\theta }$, and the stability boundary (Fig. 4) built up iteratively. Alternatively, two-parameter continuations in $K_{\theta }$ and $K_{\psi }$ can be performed on either of the Hopf bifurcations and the branch point to trace their loci in the $K_{\theta }-K_{\psi }$ plane, and this method is employed here. Plotting these continuations, shown in Fig. 6, reconstructs the stability boundary obtained in Fig. 4. Now however, the significance of each part of the boundary is known, as well as the path of each segment once inside the unstable region.

**Table 2:** Key to symbols and lines used in bifurcation diagrams

Graphic	Description	Meaning
	Solid green line	Stable equilibrium branch
	Dashed magenta line	Unstable equilibrium branch
	Solid blue line	Stable periodic solution branch (max. value of LCO)
	Dotted red line	Unstable periodic solution branch (max. value of LCO)
$\square$	Hollow square	Hopf bifurcation
$\bigstar$	Black star	Branch point bifurcation
$\CIRCLE$	Black circle	Limit point (fold) bifurcation
$\blacktriangle$	Black triangle	Homoclinic bifurcation
$\triangle$	Hollow triangle	Heteroclinic bifurcation

Considering classical aeroelasticity, two types of instability often discussed are static divergence and flutter. Static divergence is a phenomenon concerning the interaction of aerodynamic loads and internal elastic forces resulting in an exponentially growing non-oscillatory structural response. On the other hand, flutter is a dynamic phenomenon involving the interplay between aerodynamic, elastic and inertial forces resulting in an exponentially growing oscillatory response. Both types of instability can be studied mathematically through eigenvalue analysis. Static divergence is characterised by a single real eigenvalue crossing the imaginary axis, from the negative to the positive half-plane. Flutter, however, involves a pair of complex conjugate eigenvalues crossing the imaginary axis in the same way. Both types of instability are observed in this research.

**Fig. 7:**
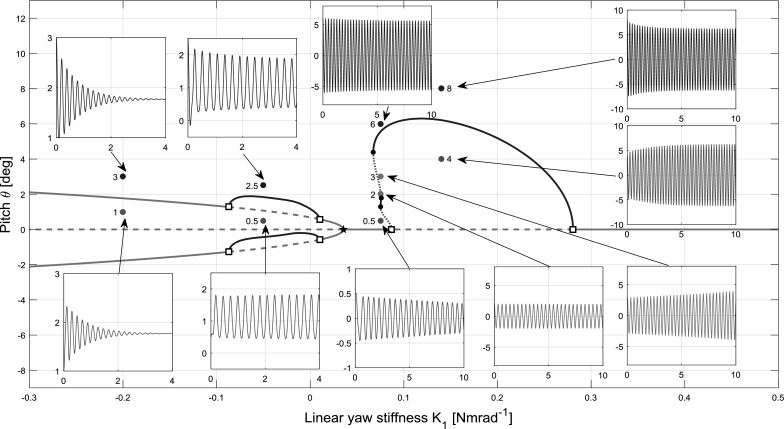
Bifurcation diagram for case 2 $(K_{\theta }=0.3)$ with $K_{1}$ as the continuation parameter, with time simulations started with a selection of $K_{1}$ values (pitch [deg] vs. time [s]). Initial conditions are shown with coloured dots. (Color figure online)

The red curved region in the bottom left corner of the diagram is defined by the location in $K_{\psi }$ of the Hopf bifurcation for a given value of $K_{\theta }$. In the same way, the blue strips that are adjacent to the axes are defined by the branch point. Recalling the definition of each bifurcation, periodic solution branches emanate from Hopf bifurcations while two additional equilibrium branches emanate from pitchfork branch points. Therefore, all points that lie within the red region have periodic solutions in $\theta$ (and in fact all the state variables). Similarly, all points that lie within the blue lobes have additional equilibrium solution branches. These bifurcations can be linked to the onset of different behaviours of the nacelle at the physical level: the periodic solutions constitute whirl flutter, while the additional equilibrium branches constitute static divergence, where the reduction in stiffness causes the nacelle to be pushed to the side by the aerodynamic forces and moments generated by the rotor.

**Fig. 8:**
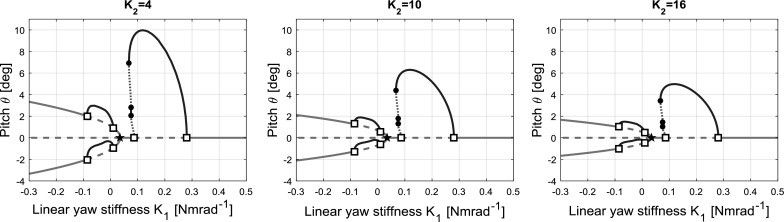
Bifurcation diagram for $K_{\theta }=0.3$, $K_{2}=[4,10,16]$, respectively, $K_{1}$ as continuation parameter

Establishing a way of describing the theoretical findings of bifurcation analysis in terms of the practically oriented language of aeroelasticity requires special care, despite the purportedly qualitative nature of both fields. The principal issue is the stability of solutions. When observed in practice, static divergence and whirl flutter are almost always immediate, irreversible “runaway” unstable motions. However, in continuation analysis, where precise “knife-edge” cases are found, both stable and unstable solution branches may be found for both equilibrium and periodic solutions, as is shown later in the paper. This leads to apparently contradictory terminology being used to describe the various behaviours observed in the model; the phrase “stable static divergence” is a contradiction in terms when viewed from the standpoint of aeroelasticity, though in the domain of bifurcation theory it refers quite clearly to a stable equilibrium branch that has emanated from a branch point. In order to preserve both the physical meaning of predicted behaviours and the insights afforded by bifurcation analysis, the terms “static divergence” and “whirl flutter” are used in direct conjunction with terms qualifying stability throughout the discussion sections of this work. A number of cases of various values of $K_{\theta }$ are selected for further bifurcation analysis and are indicated in Fig. 6.

As the Hopf and branch point are both on the equilibrium branch, which lies at zero displacement, the positions of the bifurcations do not change with the addition of any nonlinear stiffness terms. However, the dynamic behaviour outside the equilibrium branch calculated in Fig. 6 (hereafter referred to as the “main branch”) is affected by nonlinear terms.

### Cubic hardening

A nonlinear yaw stiffness profile with a $K_{2}$ value of 10 and a $K_{3}$ value of 0 was used in the cubic hardening model. To facilitate understanding, case 2 $(K_{\theta }=0.3)$ is initially considered in detail due to the variety of dynamical behaviours present. A bifurcation diagram of the pitch projection is presented in Fig. 7. The figure shows complex behaviour manifested in stable and unstable limit cycles and secondary equilibrium branches. Note that the continuation parameter is now $K_{1}$ rather than $K_{\psi }$. While the limit cycle branches illustrate the behaviour of the rotor-nacelle system encountering whirl flutter, the secondary branches emanating from the branch point bifurcation quantify the static divergence in pitch and yaw. For limit cycles, solid blue denotes stability and dashed red denotes instability. It is common practice to illustrate only the maximum positive extent of a limit cycle branch. Moreover, it is typical in bifurcation analysis to extend the continuation outside the physical range to search for any bifurcations which result in secondary branches extending back to the physical parameter range.

While bifurcation analysis is able to reveal complex behaviours of a system, the best approach is to supplement continuation with time simulations at points of interest for a fuller understanding. Time histories in pitch $\theta$ for a number of values of $K_{1}$ are also shown in Fig. 7, with different initial conditions to demonstrate the stability of limit cycles by showing convergence or divergence as relevant. From left to right, the areas of interest that are selected for time simulation are the stable static divergence branch at $K_{1}=-\,0.2$ (demonstrating convergence on approximately $1.8{^{\circ }}$), the stable flutter on the static divergence branch at $K_{1}=-\,0.05$ (demonstrating convergence on a limit cycle centred at approximately $1.2{^{\circ }}$), both stable and unstable regions of flutter on the main branch at $K_{1}=0.075$ (demonstrating divergence from a limit cycle with amplitude of approximately $3{^{\circ }}$, convergence on a limit cycle with amplitude of approximately $5{^{\circ }}$ and convergence on a limit cycle of approximately $2{^{\circ }}$) and stable flutter on the main branch at $K_{1}=0.14$ (demonstrating convergence on a limit cycle with amplitude of approximately $6.3{^{\circ }}$). All four state projections for hardening model case 2 are shown in Fig. 11 (left column) to provide a full comparison between the behaviours of each system.

**Fig. 9:**
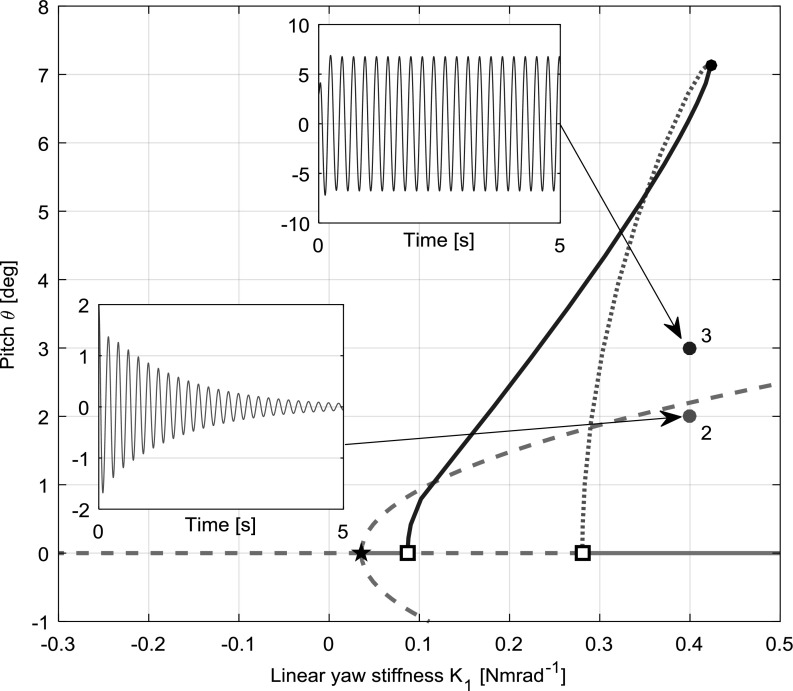
Bifurcation diagram for $K_{\theta }=0.3$, $K_{2}=-10$, $K_{1}$ as continuation parameter, complemented with time simulations started with a selection of $K_{1}$. Initial conditions are shown with coloured dots. (Color figure online)

The value of cubic stiffness coefficient $K_{2}$ used above was selected rather arbitrarily in order to effect nonlinear stiffness behaviour. It is therefore prudent also to understand the effect of the value of $K_{2}$. Bifurcation diagrams with $K_{\theta }$ set to 0.3 (as per case 2) for decreased and increased values of $K_{2}$ are shown in Fig. 8 compared to the original value of $K_{2}=10$. As is evident from the plots, increasing $K_{2}$ decreases the amplitude of both the flutter and the static divergence for a given value of $K_{1}$, due to increased structural stiffness.

It is noted that the periodic solution branch connected to the main branch always leans over the Hopf bifurcation adjacent to the branch point. As a result, a portion of the branch connecting to this bifurcation is unstable, which is present for all positive values of $K_{2}$. Furthermore, the effects of changing $K_{2}$ could also be explored for the other cases shown in Fig. 6, but this was deemed outside the scope of this paper.

### Cubic softening

Using a softening yaw stiffness profile $(K_{2}=-\,10,K_{3}=0)$, a bifurcation diagram for case 2 $(K_{\theta }=0.3)$ showing the pitch projection is presented in Fig. 9. The static divergence branches emanating from the branch point at $K_{1}=0.04$, though unstable, overhang the main branch, to the right of the Hopf bifurcation near $K_{1}=0.3$. While an unstable flutter solution emanates from this Hopf bifurcation, this branch eventually becomes stable through a limit point at approximately $K_{1}=0.42$, and both portions overhang the stable portion of the main branch (from $K_{1}=0.28$ upwards) as far as this limit point. Time simulations for selected points are shown in subplots.

A rotor-nacelle mounted on an aircraft is subject to perturbations, from manoeuvring or gusts, for example. A perturbation of the rotor-nacelle may ultimately bring it sufficiently close to either of these solution branches to experience behaviour of either type. The time simulations show that for $K_{1}=0.4$, where the main branch is stable, a pitch perturbation of $2{^{\circ }}$ provides a decaying motion, but a stable flutter LCO develops almost immediately with a perturbation of $3{^{\circ }}$. In general, a perturbation may consist of any combination of individual state perturbations (i.e. angles and angular rates). Provided sufficient angular rates, attraction to the stable flutter branch overhanging $K_{1}=0.4$ could be possible from even lower angles than $3{^{\circ }}$, and conversely larger perturbations than $3{^{\circ }}$ may converge on the main branch if the angular rate components are not sufficiently large.

**Fig. 10:**
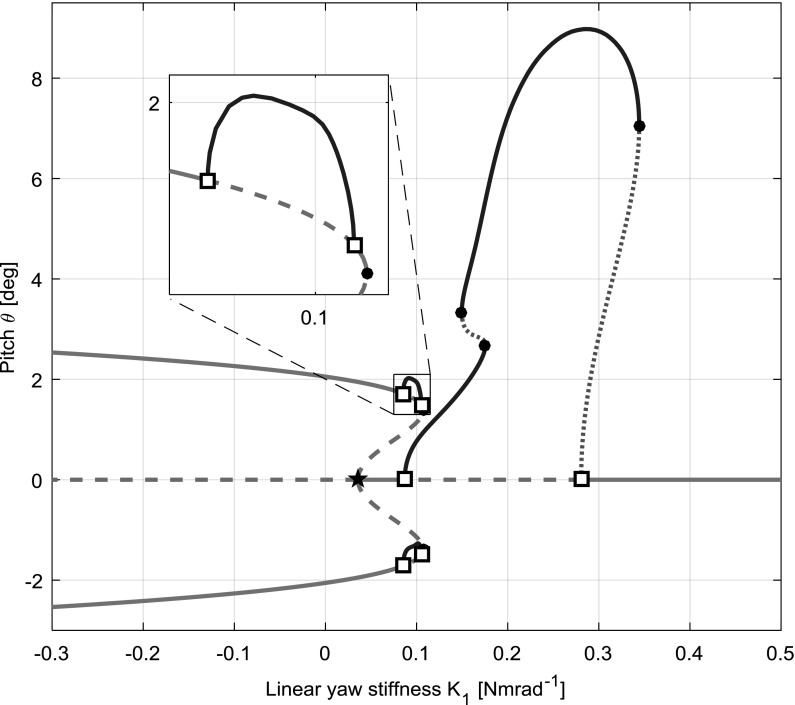
Bifurcation diagram for combined softening-hardening, case 2 $(K_{\theta }=0.3)$

The linear stability analysis is unable to predict the above result. The flutter boundary it predicts is simply the location of the Hopf bifurcation at $K_{1}=0.28$, though both flutter and static divergence behaviours are shown to exist and may be encountered for values of $K_{1}$ that lie within the stable region.

### Combined cubic softening: quintic hardening

Introducing a positive quintic coefficient $(K_{3})$ into the stiffness profile used in Sect. 4.4 allows softening effects to dominate at lower deflections and hardening effects at higher deflections. Compared with the softening model’s results, the hardening effects bend the static divergence branches back round to the left, allowing a small branch of flutter LCO’s to exist on each, as seen in the hardening model. This type of flutter is termed “secondary flutter” in the remainder of this paper to distinguish it from flutter about the main branch. The value of $K_{3}$ chosen was 350, so that the stiffness curve is close to the linear profile within the angle range of $[-10{^{\circ }},\,10{^{\circ }}]$ (see Fig. 2). Initially, the bifurcation diagram for case 2 is presented in Fig. 10.

**Fig. 11:**
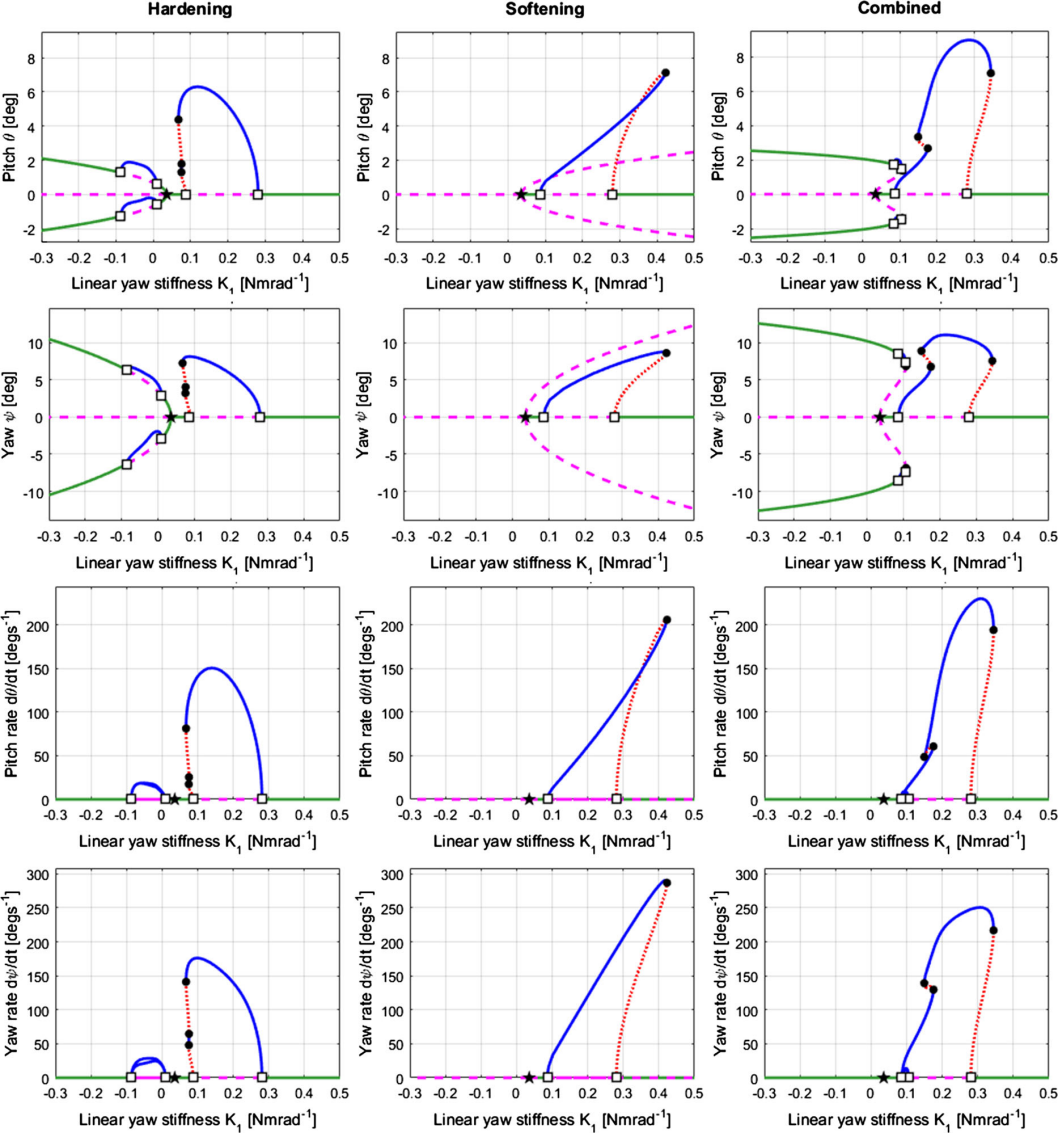
Bifurcation diagrams for case 2 $(K_{\theta }=0.3)$ in all state projections for hardening (left), softening (centre) and combined softening–hardening (right) models

**Fig. 12:**
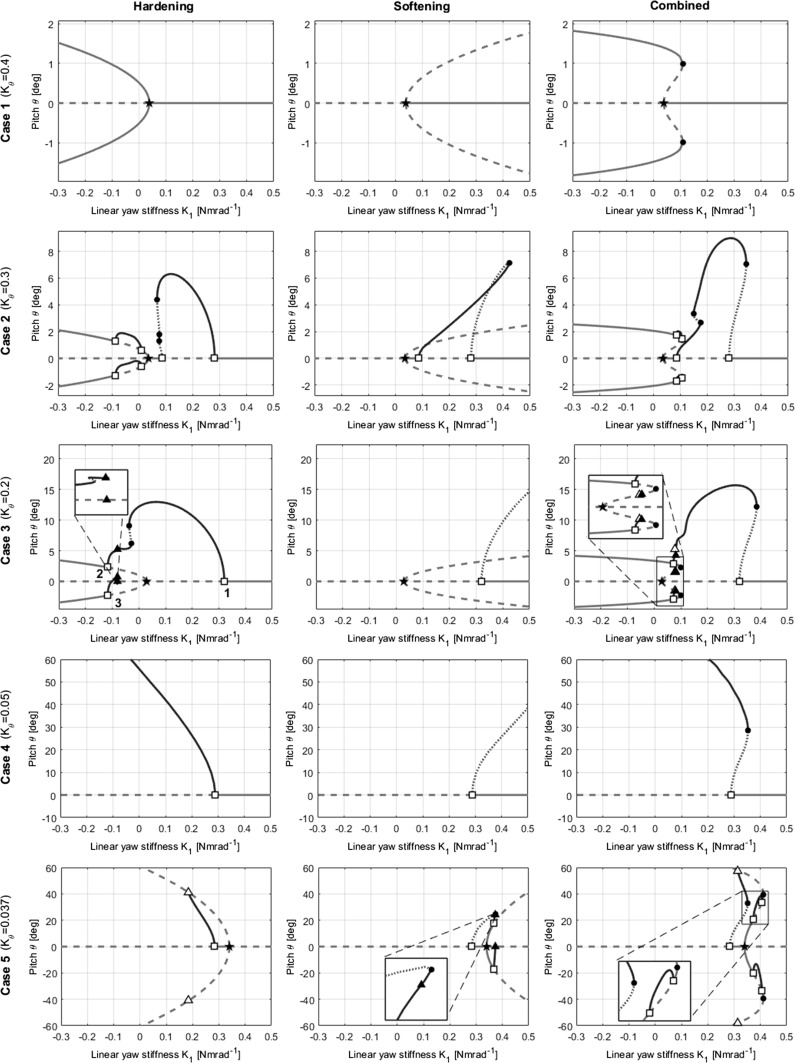
Summary of pitch projections for all cases, for hardening, softening and combined models, with $K_{1}$ as the continuation parameter

To provide a level comparison between the behaviours of each stiffness case (hardening, softening, combined), the projections for all states for case 2 are shown in Fig. 11. As before, the secondary equilibrium branches in the pitch and yaw projections (first two rows of Fig. 11) show the static divergence for a given value of $K_{1}$. As static divergence does not involve any movement by definition, these secondary branches appear to overlap the main branch in the pitch rate and yaw rate projections (last two rows of Fig. 11) in all three models, as in both branches pitch rate and yaw rate are zero. In the hardening and combined models (left and right columns of Fig. 11), each static divergence branch has its own secondary flutter LCO branch. The pitch projections for all five $K_{\theta }$ cases from all three models are summarised in Fig. 12.

Considering a given diagram from right to left, i.e. for descending $K_{1}$: case 1 $(K_{\theta }=0.4)$ shows divergence only (Fig. 12a), case 2 $(K_{\theta }=0.3)$ shows a separate region of flutter only followed by divergence (Figs. 7, 9, 10), case 3 $(K_{\theta }=0.2)$ shows flutter which eventually coexists with static divergence (Fig. 12c), case 4 $(K_{\theta }=0.05)$ shows flutter only (Fig. 12d), and case 5 $(K_{\theta }=0.037)$ shows a separate region of divergence followed by flutter (Fig. 12e). Only the projection in pitch $\theta$ is shown in Fig. 12, though projections in any of the other state variables would present the same qualitative results. Solutions above $60{^{\circ }}$ in cases 4 and 5 have been ignored as they are considered extreme non-physical behaviour.

Each of the diagrams can be cross-referenced with Fig. 6 to confirm that the bifurcations present on the main branch correspond to the extent of the unstable regions at the relevant value of $K_{\theta }$. As the value of pitch stiffness is gradually decreased, the amplitude of the limit cycles increases significantly.

Interesting to note is the complex interaction in case 3 $(K_{\theta }=0.2)$ in the hardening and combined models (Fig. 12c, left and right) that occurs between the limit cycles emanating from the main branch (Hopf 1) and those emanating from the two secondary branches (Hopfs 2 and 3). In case 3, these limit cycle branches have collided due to a homoclinic bifurcation, covered in more detail in Sect. 4.6. On the other hand, a collision between a flutter branch and a static divergence branch occurs in case 5 $(K_{\theta }=0.037)$, due to a heteroclinic bifurcation.

Regarding the results from the softening model, the bifurcations on the main branch still occur in the same left-to-right order as in Fig. 7, as nonlinear stiffness terms do not affect their location. Moreover, the regions of stability of the main branch are unaffected. However, both the static divergence and flutter branches are reversed left-to-right in the direction of their departure from the main branch. With the exception of case 5, all the static divergence branches are unstable and no secondary limit cycle branches were found to emanate from them, as seen in cases 2 and 3 of the hardening model. The crossing of the stable and unstable portions of the flutter branch in case 2 at approximately $K_{1}=0.35$ in the pitch projection without a bifurcation may seem unusual at a first glance. However, a separation between the branches can be seen in other projections (such as yaw, Fig. 11). The crossing is due only to the two-dimensional projection into the $K_{1}-\theta$ plane, and the solution branch does not cross itself within the four-dimensional state space. The flutter branches in case 3 are no longer bounded or stable as they were in the hardening model. Furthermore, the flutter branch in case 5 is now connected to the secondary flutter branches through a homoclinic bifurcation.

The values of $K_{1}$ at which the bifurcations on the main branch occur is still unchanged in the combined softening–hardening model, as is to be expected. The static divergence branches depart from the main branch in the same manner as in the softening model in terms of direction and stability, though at larger deflections (i.e. further away from the main branch) the quintic hardening overpowers the cubic softening and the branches are bent back in the direction of the hardening model’s branches.

The flutter branches in the combined model cases mainly resemble those of the hardening model cases in terms of shape; however, the regions of stability on the branches have more in common with the softening cases. This seems to be another effect of the differing dominant regions of the cubic and quintic terms. The cubic softening’s dominance at low deflections influences the direction of branch’s departure from the main branch. By contrast, the quintic hardening’s dominance at higher deflections plays a greater part in influencing the path of the branch through the state space, specifically which other bifurcations the branch is connected to. This affects the overall shape of the branch and causes resemblance of the hardening model’s diagrams. As the stability of periodic solution branches changes through limit points, it is the combination of departure direction and overall shape that influences the regions of stability along a given branch. For example, a branch that departs a bifurcation in one direction, but eventually connects to another bifurcation in the opposite direction, will have both stable and unstable portions. In contrast, if the branch spanned the two bifurcations without a change in direction and therefore a limit point, there would not necessarily have been a change in stability.

Taking a broader view of the bifurcations and branch shapes in each system allows some links to become clear. The branch points with their stable equilibrium branches in the hardening model can be directly attributed to the hardening term $(K_{2})$ in the stiffness function due to the close resemblance of the supercritical pitchfork bifurcation normal form. Similarly, the softening term present in the softening and combined models closely resembles the subcritical pitchfork bifurcation normal form.

**Fig. 13:**
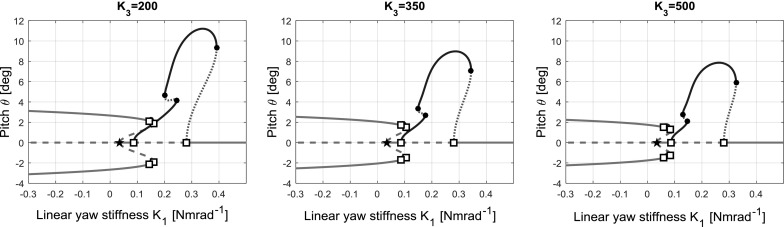
Bifurcation diagram for $K_{\theta }=0.3$, $K_{2}=-10$, $K_{3}=[200,350,500]$, $K_{1}$ as continuation parameter

In the same manner that the effect of the value of the cubic stiffening coefficient $K_{2}$ was investigated in Sect. 4.3, the effect of the value of the quintic stiffening coefficient $K_{3}$ on the combined softening–hardening model’s behaviour is investigated here. Bifurcation diagrams for increased and decreased values of $K_{3}$ are shown in Fig. 13.

The effect of $K_{3}$ is similar to the effect of $K_{2}$ in that a larger value makes for a stiffer structure than a lower value, and the effect is to restrict the extent of the static divergence branches and the amplitude of periodic solutions. As in Sect. 4.3, a more detailed investigation into the influence of $K_{3}$ could be carried out, varying other parameters such as $K_{\theta }$ and $K_{2}$, though this is deemed beyond the scope of the study presented in this article.

### Homoclinic and heteroclinic bifurcations

As the continuation parameter is varied, a portion of a limit cycle may approach a fixed point. Although the fixed point may be unstable, the vector field (as described by the differential equations of motion) in its near vicinity will be increasingly flat approaching the fixed point. Therefore, the period of the limit cycle will increase as it approaches the fixed point, reaching infinity when the collision occurs and the heteroclinic/homoclinic trajectory is created. This increase therefore can be used as an indication of the presence of such a bifurcation.

**Fig. 14:**
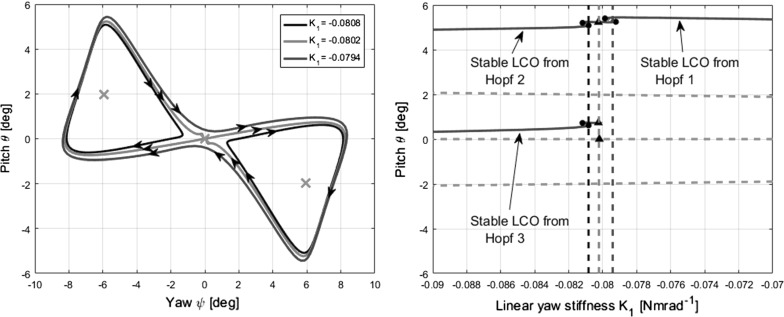
Phase portrait (left) for case 3 $(K_{\theta }=0.2)$, hardening model, showing limit cycles near the homoclinic bifurcation at $K_{1}= -0.08$. The magenta crosses indicate the three fixed points present. Enlarged bifurcation diagram (right) for cross-reference

In the hardening and combined models of case 3 $(K_{\theta }=0.2$; Fig. 12c, left and right), the behaviour of the limit cycles is explained by the heteroclinic and homoclinic bifurcations that they undergo. Taking the hardening model first, a phase portrait is shown in Fig. 14 to demonstrate how the limit cycles collide with a fixed point.

The solutions for three values of $K_{1}$ are illustrated—two limit cycles, one on either side of the homoclinic bifurcation (blue and black), and the homoclinic orbit itself (red). The various elements of the phase portrait can be cross-referenced with the excerpt of the bifurcation diagram provided on the right side of the figure. In both of these plots, the maximum (positive) amplitude of each limit cycle and the position of the fixed point branches are visible. To the left of the bifurcation point, two separate limit cycles exist (black), each about one of the static divergence branches. As $K_{1}$ increases, the innermost corner of each limit cycle nears the equilibrium at the origin—the main branch mentioned in previous sections. The limit cycles simultaneously make contact with the origin fixed point at $K_{1}= -\,0.0802$, fusing to form a homoclinic trajectory (red). Beyond this value of a $K_{1}$, a limit cycle forms and the trajectory loosens, taking on the appearance of a bow tie (blue).

**Fig. 15:**
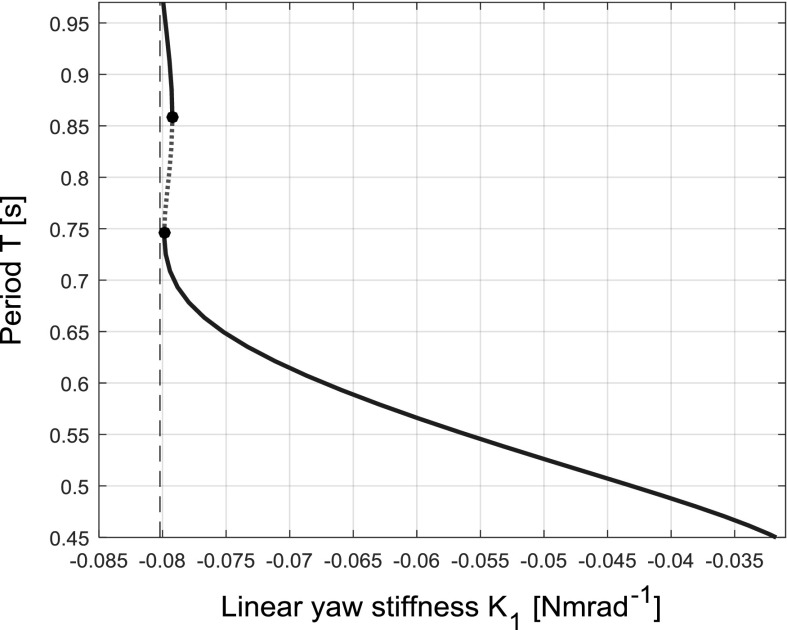
Graph of limit cycle period over a subset of the continuation parameter range shown in Fig. 12 (left), near the homoclinic bifurcation at $K_{1}= -\,0.0802$ for case 3 $(K_{\theta }=0.2)$, hardening model

The homoclinic bifurcation itself is therefore at $[\psi ,\,\theta ,\,K_{1}] = [0,\,0,\,-\,0.0802]$, as this is the point at which the two limit cycles collide and fuse. On the bifurcation diagram shown in Fig. 12c (left), the limit cycle branches are indicated by their maximum amplitude, and therefore, the secondary flutter branches seem to disappear on this hyper-plane. To indicate their annihilation via a homoclinic bifurcation, the ends of the branches are also marked with the homoclinic bifurcation symbol. The period of the larger single limit cycle approaching the homoclinic bifurcation (from the right) is shown in Fig. 15. The stability and limit points are also included for cross-referencing with Figs. 12c (left) and 14 (right). The characteristic asymptotic increase in period near the bifurcation is clearly visible.

**Fig. 16:**
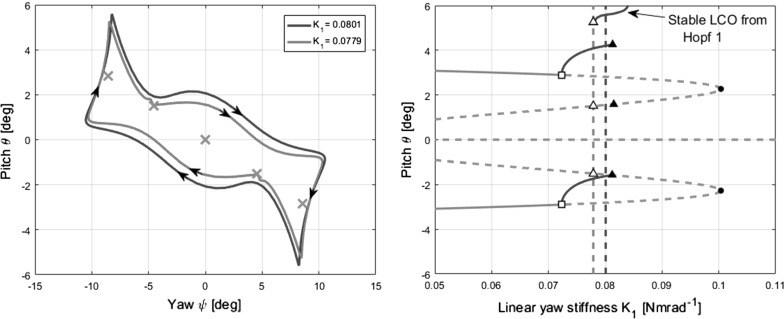
Phase portrait showing a limit cycle near the heteroclinic bifurcation at $K_{1}=0.0779$ (left). The magenta crosses indicate the five fixed points present. Enlarged bifurcation diagram (right) for cross-reference

The combined model’s results feature homoclinic bifurcations and heteroclinic bifurcation. The heteroclinic bifurcation at $K_{1}=0.0779$ is explored first. Unlike the homoclinic bifurcations seen previously, the heteroclinic bifurcation involves a heteroclinic trajectory that joins two equilibria. A phase portrait showing trajectories at and near the bifurcation is shown in Fig. 16.

**Fig. 17:**
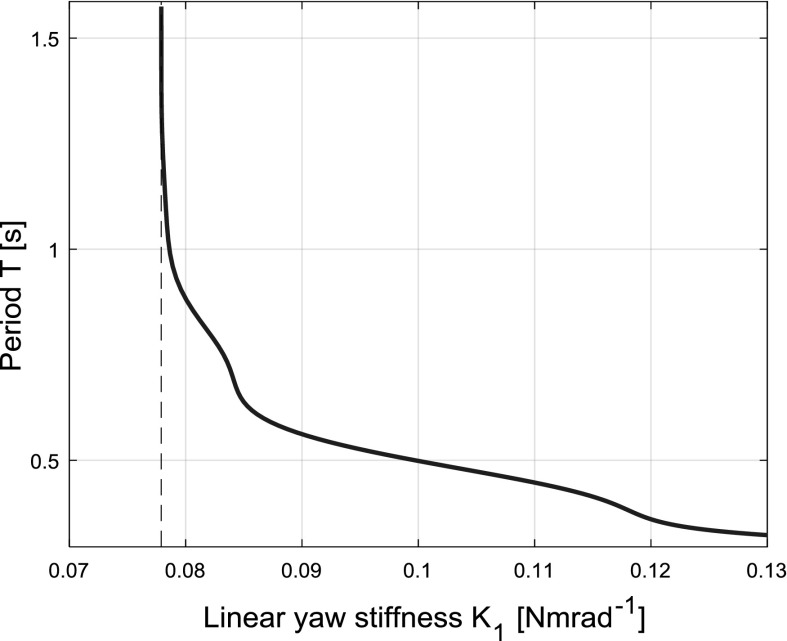
Graph of limit cycle period over a subset of the continuation parameter range shown in Fig. 12c (right), near the heteroclinic bifurcation at $K_{1}=0.0779$

The two equilibria that are joined by the heteroclinic trajectory are the inner pair of fixed points at $[\psi ,\,\theta ]=\pm \,[-\,4.5,\,1.5]$. As the complete motion strictly comprises two trajectories, one from left to right and the other vice versa, it is termed a heteroclinic cycle [31]. As the bifurcation point is approached from beneath (increasing $K_{1}$), certain corners of the limit cycle move towards the fixed points mentioned, eventually colliding with them simultaneously at $K_{1}=0.0779$. The period of the limit cycle approaching the heteroclinic bifurcation (from the right) is shown in Fig. 17.

There are also two homoclinic bifurcations at $K_{1}= 0.0828$ where the secondary flutter branches collide with the same inner pair of fixed points involved in the heteroclinic bifurcation.

Homoclinic and heteroclinic bifurcations are also visible in case 5 $(K_{\theta }=0.037)$. In the hardening model (Fig. 12e, left), the periodic branch’s maximum and minimum extents simultaneously make contact with the static divergence branches, annihilating after forming a heteroclinic cycle between the two equilibria. In the softening model (Fig. 12e, centre), the unstable flutter branch folds back to become stable at approximately $K_{1}=0.38$, and very shortly afterwards splits into two limit cycles via a homoclinic bifurcation at $[\psi ,\,\theta ] = [0,\,0]$. These two new limit cycles are secondary flutter motions about the static divergence branches. This is the same process that occurred in the hardening model for case 3 $(K_{\theta } = 0.2)$, interpreted in reverse. In the combined model (Fig. 12e, right), the main flutter branch collides with both static divergence branches simultaneously, in the same manner as in the hardening model.

### Implications for stability boundaries

As shown in Figs. 11 and 12, stable regions of the flutter branch emanating from the Hopf bifurcation on the main branch can overhang the main branch itself in the softening and combined models, meaning that flutter can be encountered despite the prediction of stability using linear analysis. In the softening model, this overhang occurs in the approximate region $0.28<K_{\theta }<0.32$. In this region, bifurcation diagrams take the form of case 2 $(K_{\theta }=0.3$; Figure 12b, centre). That is, a stable flutter branch exists but is connected only to the main branch, and the static divergence branches each have a secondary flutter branch about a small portion of them. The region is bounded by the existence of all the necessary bifurcations; at approximately $K_{\theta }=0.28$ the left-most Hopf and the pitchfork collide and the left-most Hopf annihilates as detailed in Sect. 4.5. For lower values of $K_{\theta }$, the periodic branch no longer has a second main branch Hopf bifurcation to fold back to, and therefore while it continues to overhang the main branch, it does not contain any stable regions (Fig. 12c–e, centre). The main branch is, however, overhung by two unstable equilibrium branches, which leads to a divergence if the system strays from the main branch.

**Fig. 18:**
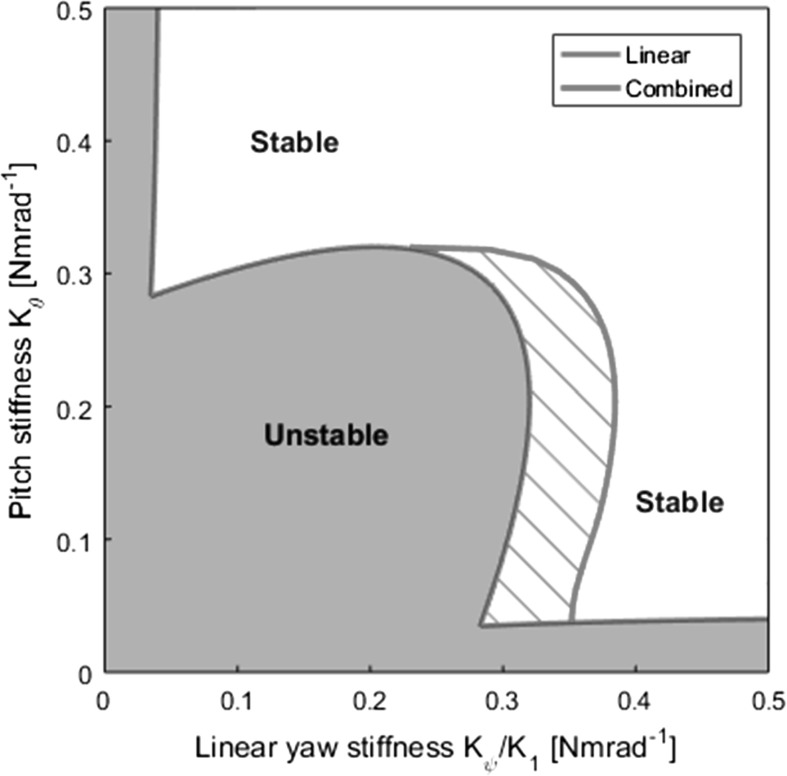
Additional unstable region area for the combined model (red) compared to the original linear prediction (grey). (Color figure online)

In the combined model, stable overhang of the main flutter branch exists for a much greater range of $K_{\theta }$. Overhang exists from $K_{\theta }=0.32$ downwards as in the softening model (Fig. 12b, right). However, after the left-most main branch Hopf bifurcation has collided with the branch point at approximately $K_{\theta }=0.28$, stable portions of flutter branch still overhang a stable portion of the main branch. Continuing to descend in $K_{\theta }$, this overhang exists until the static divergence region near the $K_{1}$ axis is met. Here, at $K_{\theta }=0.037$, the main branch rightward of the branch point does experience stable flutter branch overhang (albeit connected to the static divergence branches), though the main branch itself is unstable (Fig. 12e, right).

A revised stability boundary accounting for the rightward reach of any overhanging flutter branch with a stable portion can be generated. This can be achieved either through iterated one-parameter bifurcation over a variety of $K_{\theta }$ values, or through two-parameter continuation of the right-most limit point found on the flutter branch to trace its path in $K_{\theta }$ and $K_{1}$ simultaneously. Such a revised stability boundary for the combined model is shown in Fig. 18. The original linear model boundary and the enclosed unstable region are shown in grey. The additional unstable area due to the aforementioned overhang phenomenon in the combined model is shown in red. The boundaries are mostly coincident though the overhang extends the region of instability to the right of the Hopf loci.

## Conclusions

This article has demonstrated the use of continuation and bifurcation methods to provide nonlinear dynamic analysis of a basic rotor-nacelle system model. Both linear and nonlinear stiffness profiles were used for the yaw degree of freedom through addition of cubic and quintic stiffening terms. The cubic stiffening behaviours investigated were softening and hardening, and quintic hardening was used in conjunction with cubic softening to create a combined softening–hardening model. Stability analysis methods were described and employed for both linear and nonlinear models. Bifurcation diagrams were generated for a number of pitch stiffness cases, in each of the hardening, softening and combined models. It was shown that in the softening and combined models, whirl flutter was possible in regions where linear analysis would have predicted stability, due to stable portions of flutter branches overhanging stable main branches. A revised stability boundary based on this phenomenon was generated for the combined model, where this phenomenon exists over the greatest range of pitch stiffness. Where whirl flutter does not cause the loss of an aircraft, oscillations induced by whirl flutter mechanisms present a fatigue hazard to aircraft nacelle mounts.

The study showed that the introduction of basic and smooth polynomial stiffness profiles into the rotor-nacelle system produced very complicated dynamics. These dynamics were manifested in the coexistence of multiple equilibrium and periodic branches, as well as the various types of bifurcation, namely fold, Hopf, branch point, homoclinic and heteroclinic. These complex behaviours could not have been predicated without proper nonlinear analysis methods such as continuation and bifurcation methods.

These observations show that nonlinear aspects of a system may have a significant impact on its dynamic behaviour, particularly where stability is a focus. It is therefore advisable to model nonlinear aspects where possible and to employ proper nonlinear analysis techniques, allowing informed system design. Stability boundaries generated should also take into account the coexistence of dynamic behaviours over parameter regions where equilibrium branches are stable. Given the likely proliferation of continuation methods and bifurcation analysis in aircraft certification, proper characterisation of aircraft materials and sub-systems is crucial, and nonlinear stiffness profiles should be used in full nonlinear models for any dynamic analysis conducted. Where analytical functions cannot be fit to material or sub-system stiffness profiles, a table-based approach could be used.

Going forward, the model used in this paper is to be developed further as part of an incremental approach. Refinements to existing aspects, such as unsteady or nonlinear aerodynamics, are to be made. Additionally, new features will also be incorporated, such as the influence of the drivetrain and blade flap and lag dynamics.

## Abbreviations

FW: Forward Whirl
BW: Backward Whirl
ADYN: Advanced european tiltrotor DYnamics and Noise
LCO: Limit Cycle Oscillation
